# Evaluation of the Performance of Grouting Materials for Saturated Riprap

**DOI:** 10.3390/ma6125713

**Published:** 2013-12-06

**Authors:** Daehyeon Kim, Sinkyu Jung, Kyungsub Cha

**Affiliations:** 1 Department of Civil Engineering, Chosun University, Gwangju 501-759, Korea; E-Mail: free_titan@hanmail.net; 2 Daewoo Construction Co., Seoul 110-713, Korea; E-Mail: kyungsub.cha@daewooenc.com

**Keywords:** injection test, impermeability, grout, saturated riprap

## Abstract

In this study, four types of grout were developed to evaluate the effect of grouting of saturated riprap layers on ground water flow. The developed types of grout are divided into a quick-setting type and a general-type, and also into high and low viscosities. A number of grout tests were performed in a model acrylic chamber, 0.4 m in diameter and 2.0 m in length, for visual observation of injection. To reproduce the field flow condition of the saturated riprap layers (approach flow), the grout tests were carried out at 0 cm/s and 100 cm/s for the flow speed and 10 L/min for the grout injection speed after installing a flow injection opening on the lower part of the chamber. Based on the results of the grout tests, the injection of each grout in the saturated riprap layers was examined to find out the most effective grout.

## 1. Introduction

Recent grouting methods using liquids have been generally applied to improve the ground. Some of the grouts include cement suspension, half solutions in which cement is mixed with at least one type of grout, and mortar. However, most types of grout experience limited permeation of the grout, grout loss due to long setting times, difficult application, difficulty in grout supply, and lowered durability due to grout leaching. Accordingly, it is necessary to repair the ground within 5–10 years after grout application.

The filling performance of the grouting in the riprap layers can deteriorate due to ground water flow. However, this issue has not been fully studied yet either in Korea or globally, and methods for the correction of poor fillings have not been suggested. The method of applying grout to match the ground water flow has the issue that most structures, for example, reservoirs or sea walls, are grouted and afterwards the grout is lost along the path of reserved water or the scale of voids gradually increases in size, resulting in leaks in the structures. Korean researchers have studied various types of grout related to the method, for example, of reinforcing soft ground or building foundations. However, further studies are required in relation to the varieties of grout for riprap layers and aggregate layers. Prior studies in each field are described below.

Achievements of developed grout include cement-, liquid- and suspension-based grout used in order to reinforce the ground. The aforementioned grout types have the disadvantage of being washed away with water and thus fail in achieving the purpose of grout application where the grout is used for saturated ground, for example, dams, embankments, and sea walls. Another issue is that harmful substances from the grout are added to ground water and sea water which causes great concern for the environment where the grout leaches into the ground water system.

Kim [1] studied the characteristics of ultra fine cement for improving the ground and tested injection features and toxicity depending on the particle diameter of the cement grout. Yu [2] *et al*. tested the optimal mixture of permeation grouting to examine injection features of grout depending on specimens. Maeng [3] improved the strength of the grout used for filling structures, and used cement grout with controlled hydration speed to test permeability. Kim [4] studied alkaline silica sol to address the weakness of water glass, and Lee [5] studied flow changes in riprap layers. As described above, a lot of studies have been undertaken examining grouts. However, most studies are related to the permeability of small voids in the ground. In other countries, most researchers have basically studied the effects of applied grout depending on the grout element (Yang Ping, [6]), and the grout applied to riprap layers to prevent erosion of abutments and bridge substructure (Luara G. Girard, [7]).

This study aims to examine the effect of applied grout for the condition of flow by mixing an expansion agent, an anti-washout agent, and a superplasticizer in order to lower leaching and segregation which occurs when a liquid and cement suspension are used.

## 2. Field Condition and Injection Test

### 2.1. Field Condition

**Figure 1:**
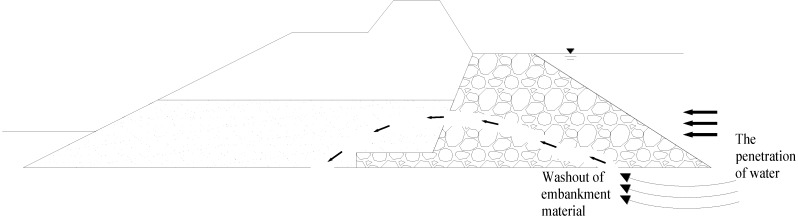
Schematic field conditions.

As shown in Figure 1, sea water and flowing water permeated into the lower part of a structure composed of riprap layers, for example, embankments and dams, causes earth and sand to leach through the riprap layers and the dams and structures thus to collapse. This results in all sorts of accidents and damages. It is also necessary to build cut-off walls by applying grout to the riprap layers, but riprap layers with large voids are saturated with fresh water or sea water. The riprap layers even have a flow which causes the grout to wash out. It is thus impossible to effectively build cut-off walls, as the walls cannot assume their role.

The flow in the riprap layers was measured to be approximately 1 cm/s. However, the approach flow was set at about 100 cm/s in the injection test so as to be conservative. The injection speed applied in grouting the riprap layers was set as approximately 10 L/s in the injection test.

### 2.2. Grout Mixture Ratio, Specimen and Tester

Table 1 illustrates the ratios of mixing materials injected in the test; superplasticizer was added to improve flowing capability in the saturated riprap layer in order to enhance the injection workability. It was attempted to find the optimum injection materials with less washout and with no segregation in the riprap layer with the flow for filling voids thereby cutting off water permeation.

**Table 1:** Mixing ratio of the injected materials (25 kg).

Classification		OPC(kg)	Special OPC * (kg)	Lime (kg)	Sand (kg)			Antiwashout material (g)	Inflating agent (g)	Superplasticizer (g)	Water (kg)
					No.4	No.5	No.6				
General type	Low viscosity	8.75	–	4	5	6	1.25	57.5	125	131.25	5.75
	High viscosity	8.75	–	4	5	6	1.25	75	125	131.25	5.75
Quick-setting type	Low viscosity	7.61	1.14	4	5	6	1.25	57.5	125	131.25	5.75
	High viscosity	7.61	1.14	4	5	6	1.25	75	125	131.25	5.75

* Note: Special OPC is a type of OPC with CAC, Gypsum and C12A7.

**Figure 2:**
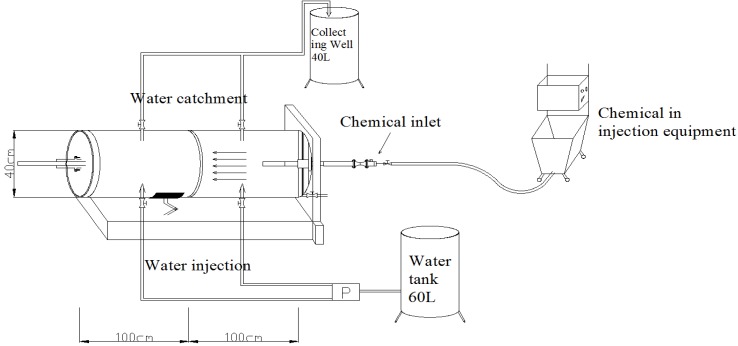
Schematic grouting and chemical injection system.

**Figure 3:**
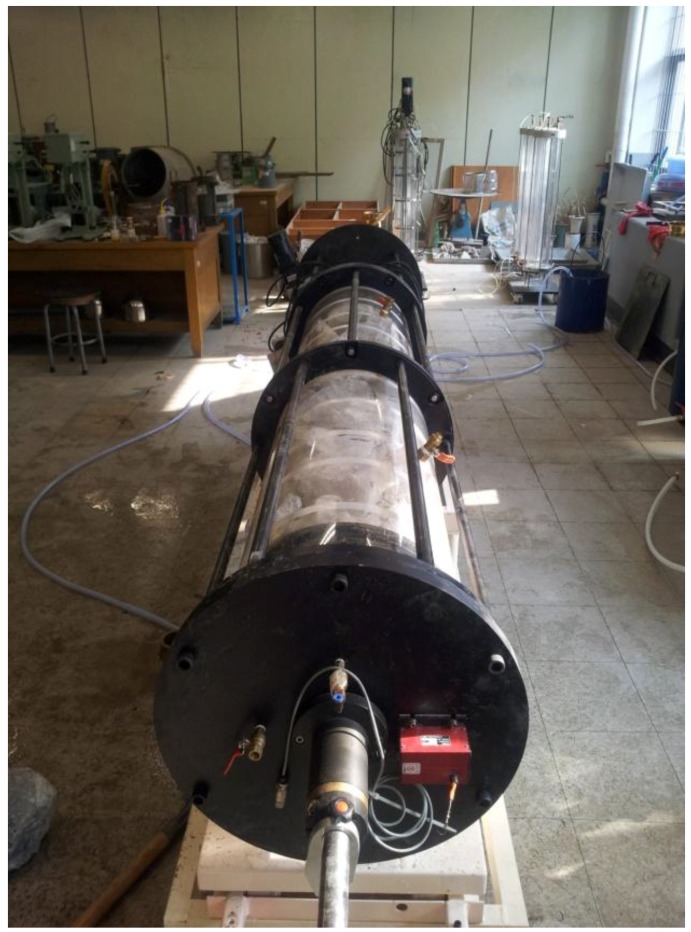
Grouting system.

For the test, a model chamber Diameter (40 cm) × Length (200 cm) was produced to reproduce a similar field condition for grouting together with an injector as shown in Figure 2. Round ripraps (diameter: 20–30 cm) were used in the test. For the flow condition, two flow valves were installed on both sides to implement a lateral flow. A flow meter was used to measure the flow as well as the volume of water flowing into the model chamber. Figure 3 shows the produced test system.

### 2.3. Method of Injection Test

The system for testing the application of grout used in the test is composed of two acrylic cylinders of diameter 0.4 m and length 1.0 m, 12 rods for connecting the two acrylic cylinders, two connection rings and an upper plate. It is possible to lift the grouting system by approximately 45° and this was intended to facilitate the test when the riprap layer was filled.

**Figure 4:**
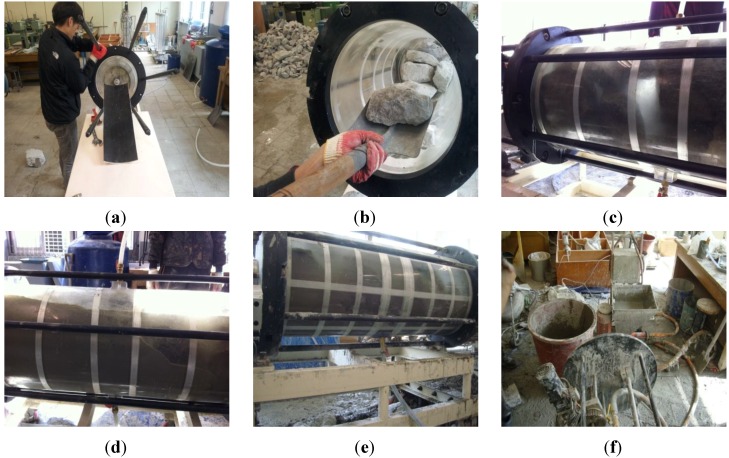
Process of grouting. (**a**) assemble the system; (**b**) fill the system with riprap; (**c**) saturated riprap layer; (**d**) inject mixed grout; (**e**) speed of moving fluid; (**f**) injection system .

Figure 4 shows the process for the grouting test. As shown in Figure 4a,b, the grouting equipment was assembled to fill the acrylic cylinders with riprap. After finishing the riprap filling, the riprap was saturated before injection of the grout material through the lower flow inlet as shown in Figure 4c. The optimally mixed grout was then finally injected as shown in Figure 4d. Injection of the grouts and flow of water were done simultaneously. For the injection speed of 10 L/min, it took about 30–35 min to maintain the flow of water, while, for the injection speed of 10 L/min, about 15–20 min. The porosity of riprap for the test was based on approximately 55% by calculating approximately 130 L of water after fully filling the riprap from the total volume of 226 L of required water.

Although the expansion time is a maximum of 3 h after expansion begins, the injection should be carried out as quickly as possible because the expansion effect is not achievable post-injection if a large portion of expansion has occurred during the mixing and injection. When the injection of a given amount of mixed grout was finished, we immediately measured the injection circumference and again 3 h after the first measurement. The speed of moving fluid and the injection system can be seen in Figure 4e,f, respectively.

## 3. Results of Injection Test 

### 3.1. Measuring Circumference of Injection and Formed Bulb

**Figure 5:**
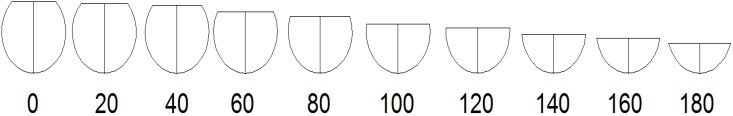
Method of measuring circumferences of injected grout.

Figure 5 shows the circumferences of the developed filling material visually measured by marking the 2 m-long acrylic cylinder at 0.2 m-intervals. The sectional circumferences are based on the measurements. The expanded circumference of the injected grout was obtained by measuring the circumferential length of the acrylic cylinder every 20 cm with the method of average end areas and then totalizing the sum of measured circumferences of intervals/2 × 20 cm for each interval to evaluate the expanded circumferences. The expansion ratio represents a difference of the expanded circumferences after injection around the expanded circumferences 3 h after injection.

The time for measuring the circumferences was set as directly after injection and 3 h after injection. The length of measured circumferences was recorded in tables and graphs to determine the expansion of the grout and the level of fluidity.

**Figure 6:**
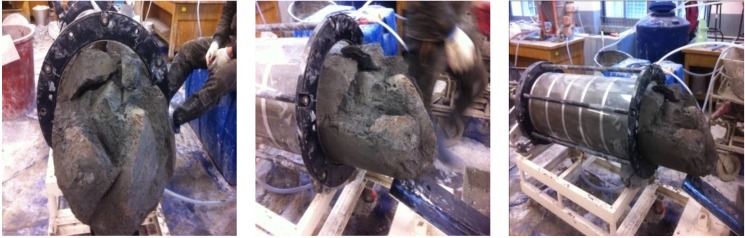
Bulb formation.

Figure 5 shows the formed bulbs observed in the initial test. Figure 6 shows the grout that remained after removing the first acrylic cylinder between two divided acrylic cylinders. In the initial test, the grout was cured for approximately 22 h after injection and the circumference measured. However, it was later changed to 3 h after injection when the grout expansion was almost finished due to acrylic deformation and damage on removal. Quick-setting and low viscosity were identified, and the quick-setting type formed bulbs within 24 h which were firm enough not to be easily broken, as shown in Figure 6.

### 3.2. Results of Circumferential Measurement for Injected Material

**Figure 7:**
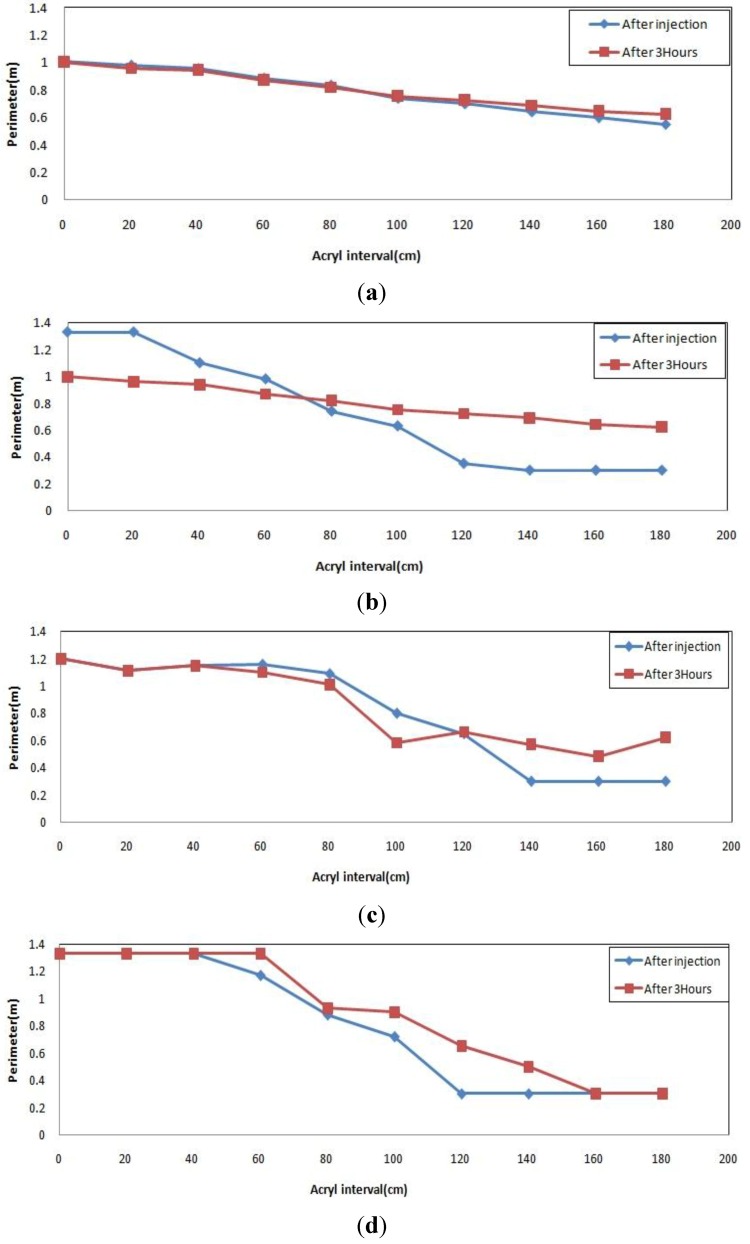
Injection pattern of general type material. (**a**) low viscosity (flow 0, injection 10); (**b**) low viscosity (flow 100, injection 10); (**c**) high viscosity (flow 0, injection 10); (**d**) high viscosity (flow 100, injection 10).

For the general type injection material, Figure 7a shows similar patterns for right after injection and 3 h after injection resulting in small expansion and low fluidity. In Figure 7b, it was impossible to identify the level of expansion because a lot of grout flowed 3 h after injection. Meanwhile, Figure 7c,d shows almost similar graph patterns, which have a gap between the two graphs. Small expansion was thus observed after some time at the high viscosity.

**Figure 8:**
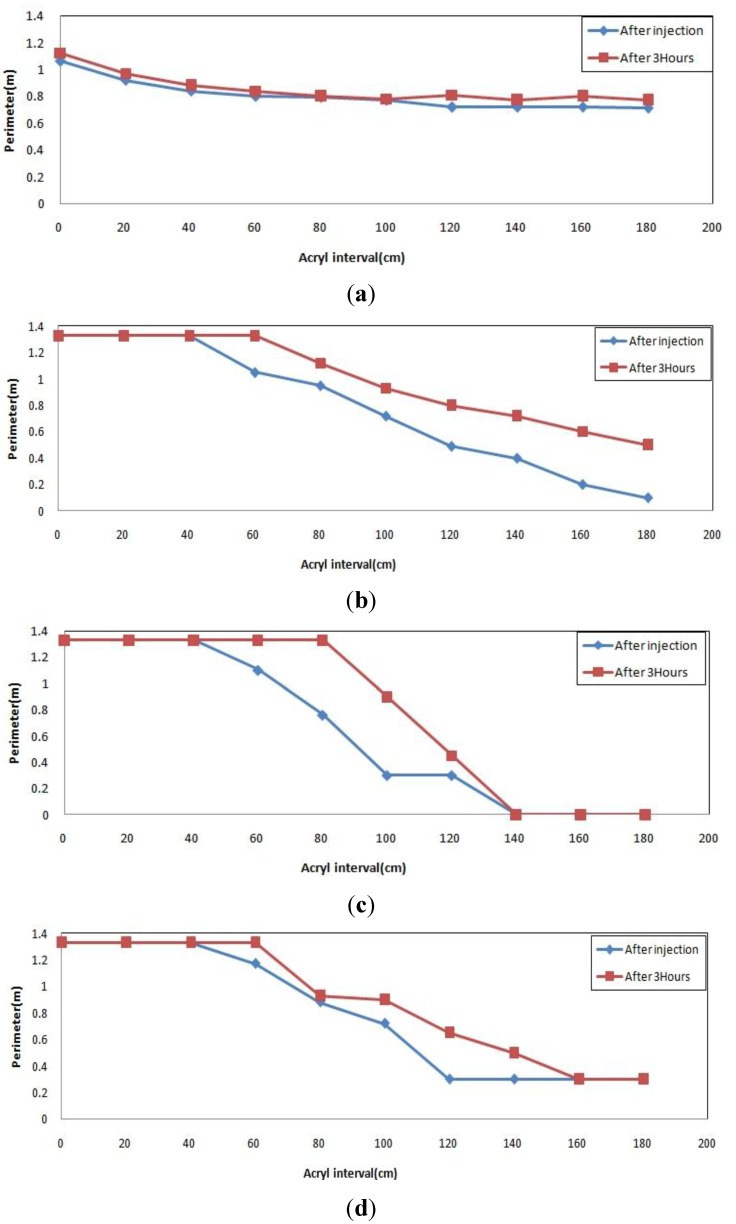
Injection pattern for quick-setting type material. (**a**) low viscosity (flow 0, injection 10); (**b**) low viscosity (flow 100, injection 10); (**c**) high viscosity (flow 0, injection 10); (**d**) high viscosity (flow 100, injection 10)

Figure 8a–d shows a pattern for the quick-setting injection material which fully filled the injection inlet right after injection, and was gradually pushed away and gradually flowed down over time. The measurement 3 h after injection revealed a higher position than the position right after injection thus indicating grout expansion over time. Higher expansibility was observed at the high viscosity.

### 3.3. Results of Circumferential Measurement Depending on Flow

Two flow conditions and two conditions over time based on the measured circumferences listed in Table 2 and Table 3 are plotted in one graph to evaluate the effect of flow on grout injection.

**Figure 9:**
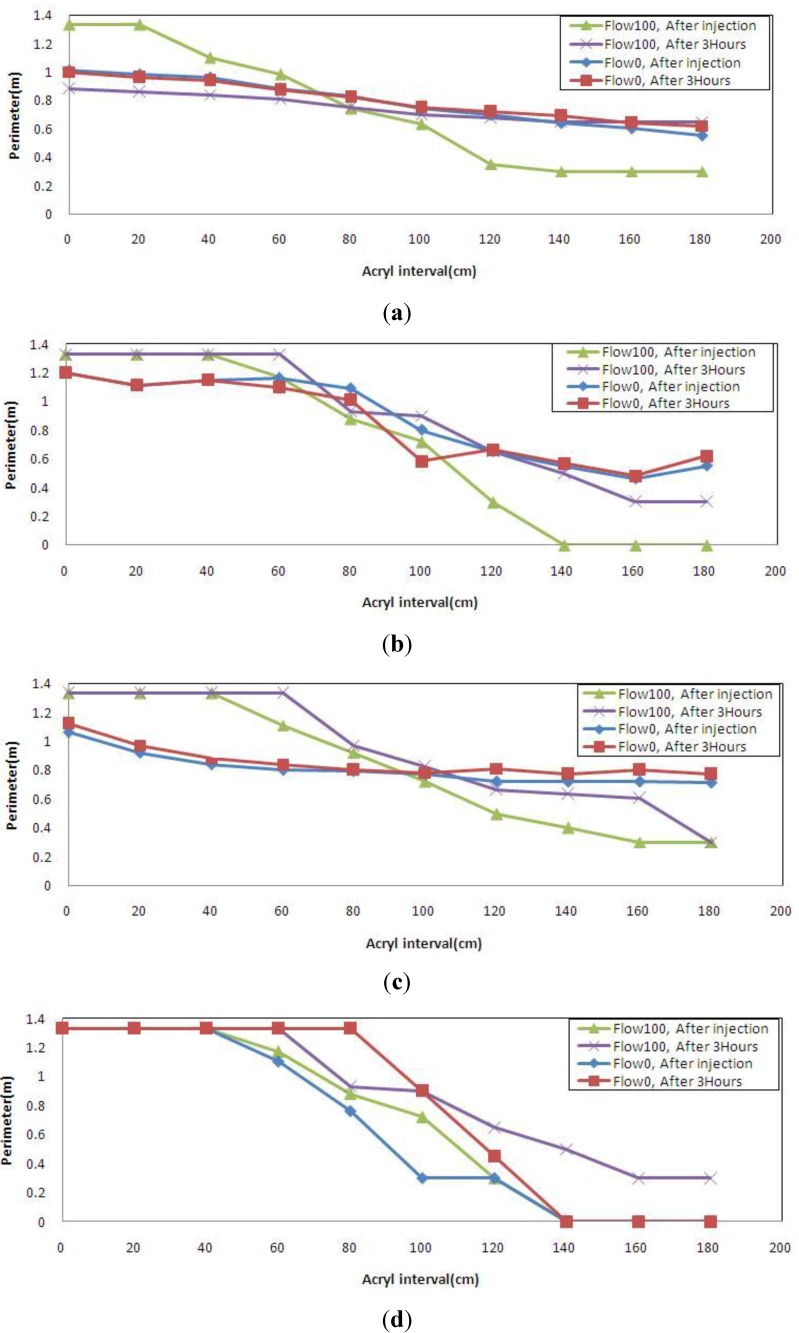
Injection pattern depending on approach speed. (**a**) general low viscosity type; (**b**) general high viscosity type; (**c**) quick-setting low viscosity type; (**d**) quick-setting high viscosity type.

Figure 9a for the general low viscosity type shows a similar pattern for after injection and 3 h after injection with no flow. However, the grout shows a pattern of flowing 3 h after injection with flow. Figure 9b,c shows a similar pattern for after injection and 3 h after injection, and also little expansibility with no flow. It is noted that in the case of water flow, the grout tends to expand rather than that it flows down. Figure 8d for the quick-setting high viscosity type shows initial full filling and then a constant flow of gradual flowing with regard to flow, and a given level of expansion with and without flow.

**Table 2:** Length measured for general type material.

Type	Materials	Injection speed (L/min)	Flow (cm/s)	Measurement time (h)	Interval for measuring circumference of injected material (m)									
					0	0.2	0.4	0.6	0.8	1.0	1.2	1.4	1.6	1.8
General Type	Low viscosity	10	0	After injection	1.01	0.98	0.96	0.88	0.83	0.74	0.70	0.64	0.60	0.55
				After 3 h	1.00	0.96	0.94	0.87	0.82	0.75	0.72	0.69	0.64	0.62
			100	After injection	1.33	1.33	1.1	0.98	0.74	0.63	0.35	0.3	0.3	0.3
				After 3 h	0.88	0.86	0.84	0.81	0.75	0.7	0.68	0.65	0.65	0.65
	High viscosity	10	0	After injection	1.2	1.11	1.15	1.16	1.09	0.8	0.65	0.55	0.46	0.55
				After 3 h	1.2	1.11	1.15	1.10	1.01	0.81	0.66	0.57	0.48	0.62
			100	After injection	1.33	1.33	1.33	1.17	0.88	0.72	0.3	0	0	0
				After 3 h	1.33	1.33	1.33	1.33	0.93	0.9	0.65	0.5	0.3	0.3

**Table 3:** Measured length for quick-setting type grout.

Type	Grout	Injection speed (L/min)	Flow condition (cm/s)	Measurement time (h)	Interval for measuring circumference of injected material (m)									
					0	0.2	0.4	0.6	0.8	1.0	1.2	1.4	1.6	1.8
Quick-setting Type	Low viscosity	10	0	After injection	1.06	0.92	0.84	0.80	0.79	0.77	0.72	0.72	0.72	0.71
				After 3 h	1.12	0.97	0.88	0.84	0.80	0.78	0.81	0.77	0.80	0.77
			100	After injection	0.95	0.95	0.93	0.75	0.73	0.68	0.63	0.54	0.5	0.3
				After 3 h	1.13	1.09	0.99	0.88	0.88	0.82	0.78	0.7	0.7	0.6
	High viscosity	10	0	After injection	1.33	1.33	1.33	1.1	0.76	0.3	0.3	0	0	0
				After 3 h	1.33	1.33	1.33	1.33	1.33	0.9	0.45	0	0	0
			100	After injection	1.33	1.33	1.33	1.11	0.92	0.72	0.49	0.4	0.3	0.3
				After 3 h	1.33	1.33	1.33	1.33	0.97	0.82	0.66	0.63	0.6	0.3

An analysis reveals that the general low viscosity type was slightly affected by flow, the general high viscosity type, the quick-setting low viscosity type and the quick-setting high viscosity type were not nearly affected by flow.

### 3.4. Analysis of Grout Expansibility 

Measuring circumferences in this study aims to examine flowing patterns, and thus to evaluate grout expansibility. Table 4 lists the result of grout expansibility. The amount of injected grout was calculated with the product of the circumference of each section measured with the same condition of length 0.2 m. The grout expansibility was calculated by comparing the amount of injected grout for right after injection with that for 3 h after injection.

Expansibility listed in Table 4 is the highest 12.42% in the general high viscosity type for flow 100/injection speed 10, and the highest 24.13% in the quick-setting high viscosity type for flow 100/injection speed 10. The graph shows a slightly different pattern, but grout expansibility was higher in the high viscosity grout than the low viscosity grout. It turned out that the expansibility of the quick-setting high viscosity was large enough to fill the gaps between the ripraps.

**Table 4:** Expansibility of grout measured over time.

Grout (Flow/Injection)	Measurement time (h)	Injection quantity	Expansibility (%)	Grout (Flow/Injection)	Measurement time (h)	Injection quantity	Expansibility (%)
General low viscosity type (0/10)	0	137.9	1.92	Quick-setting low viscosity type (0/10)	0	141.2	5.80
	3	140.6			3	149.9	
General low viscosity type (100/10)	0	120.6	8.64	Quick-setting low viscosity type (100/10)	0	120.2	19.44
	3	132			3	149.2	
General high viscosity type (0/10)	0	138.1	5.75	Quick-setting high viscosity type (0/10)	0	102.4	23.24
	3	146.5			3	133.4	
General high viscosity type (100/10)	0	132.6	12.42	Quick-setting high viscosity type (100/10)	0	131.4	24.13
	3	151.4			3	173.2	

### 3.5. Evaluation of Segregation

**Figure 10:**
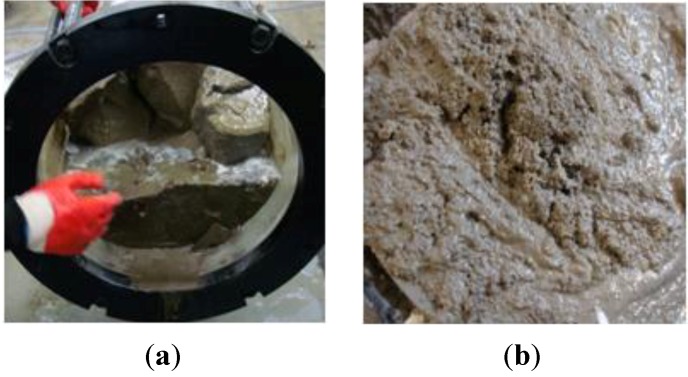
Segregation. (**a**) entrance to the precipitation of cement; (**b**) segregated inlet sand.

The evaluation of no segregation aims to fill the riprap voids with injected grout in the saturated riprap layers and to test whether the grout is not segregated in water so as to function as grout. Generally, the specific gravity of the fine aggregate is 2.5 to 2.65, and the specific gravity of cement is 3.10–3.15. If the grout was segregated and cement was accumulated at the end, it was assumed that this created a difference in the specific gravity between the front and the end to result in grout segregation. Figure 10 shows segregated grout.

Table 5 shows a great deviation 30.48% between the front and the end in the saturated riprap layer for mortar. It was identified that grout segregation was a factor of a great specific gravity difference between the front and the end. Because the test of sampling sediment of the cement paste revealed no specific gravity difference between the front and the end, the same specific gravity was achieved. The developed grout was identified as not showing a great deviation because of no material segregation thanks to the no-segregation agent. No-segregation agent is an admixture that prevents segregation of the compositions of the grout material such as cement and mortar after grouting is done.

**Table 5:** Specific gravity measured.

Gravity material	Front	End	Deviation (%)
Mortar (Flow 0, Injection 10)	2.161	3.104	30.38
Cement (Flow 0, Injection 10)	2.503	2.503	0
General low viscosity (Flow 0, Injection 10)	2.506	2.568	2.41
General High viscosity (Flow 100, Injection 10)	2.504	2.460	1.76
Quick-setting low viscosity (Flow 100, Injection 10)	2.506	2.612	4.06
Quick-setting high viscosity (Flow 0, Injection 10)	2.515	2.496	0.75
Quick-setting high viscosity (Flow 100, Injection 10)	2.343	2.398	2.29

## 4. Summary and Conclusions 

Based on the analysis of the injections tests on different types of grouts, the following conclusions can be drawn.

- Examination of grout flow and expansibility over time revealed that the general low viscosity type grout exhibited high fluidity and slow setting and is thus ideal for areas with a wide injection range. The general high viscosity type grout exhibited low fluidity, but high expansibility, and is thus ideal for ground with great voids and no flow like riprap layers. The quick-setting low viscosity type and the high viscosity type also showed similar patterns to the general low viscosity type and the high viscosity type, but were analyzed as ideal for saturated riprap layers because of quick-setting.
- Evaluation of grout flowing and expansion patterns depending on flow (0 cm/s, 100 cm/s) revealed that the general low viscosity type was slightly affected by flow resulting in grout flowing, but was analyzed as not affected by flow in other tests.
- Assessment of grout expansibility revealed that the highest expansibility of the general high viscosity type for flow 100/injection speed 10 was 12.42% and the highest expansibility of the quick-setting high viscosity type for flow 100/injection speed 10 was 24.13%. An analysis revealed that high viscosity grout had higher expansibility than low viscosity grout, and the expansibility of the quick-setting type was twice as high as the general type.
- Evaluation of no segregation of developed grout through the specific gravity test revealed a material segregation 2.161 for the front and 3.104 for the end of the conventional injection material mortar. However, the developed material used in this study had a slight deviation not to cause material segregation and is thus an ideal grout for riprap layers with great voids.
- The expansibility of the quick-setting high viscosity was large enough to fill the gaps between the ripraps, and the washout of the grout in the ripraps with ground water flow was negligible. This clearly indicates that the quick-setting high viscosity type showed a satisfactory performance in the grouting of the saturated riprap layer with water flow.

## References

[1] Kim J.C. (1999). A study on Injection Characteristics of Cement. Ph.D. Thesis.

[2] Yu H.K., Bu S.A., Han S.J., Kim S.S. Optimum Mixing Design. Proceedings of KSCE Conference.

[3] Maeng S.S. (2009). Particle Limit on the Grouting of Embankment. MSCE Thesis.

[4] Kim H.Y. (1999). A Study on Characteristics of Silica-Cement. Ph.D. Thesis.

[5] Lee H.W. (2005). Infiltration Characteristics for Embankment. Ph.D. Thesis.

[6] Yang P., Peng Z.B., Tang Y.Q., Peng W.X., He Z.M. (2008). Penetration grouting reinforcement of sandy gravel. J. Cent. South Univ. Technol..

[7] Girard L.G., Clopper P.E. Integration European partially grouted riprap for stream stability and bridge scour protection. http://www.ieca.org/membersonly/cms/content/Proceedings/Object338PDFEnglish.pdf.

